# From formulation to structure: 3D electron diffraction for the structure solution of a new indomethacin polymorph from an amorphous solid dispersion

**DOI:** 10.1107/S2052252524008121

**Published:** 2024-08-28

**Authors:** Helen W. Leung, Royston C. B. Copley, Giulio I. Lampronti, Sarah J. Day, Lucy K. Saunders, Duncan N. Johnstone, Paul A. Midgley

**Affiliations:** a Department of Materials Science and Metallurgy University of Cambridge 27 Charles Babbage Road CambridgeCB3 0FS United Kingdom; b GSK R&D, Gunnels Wood Road, StevenageSG1 2NY, United Kingdom; c Beamline I11 Diamond Light Source Didcot OxfordOX11 0DE United Kingdom; Istituto Italiano di Tecnologia, Italy

**Keywords:** indomethacin, amorphous solid dispersions, drug development, 3D electron diffraction, polymorphism, structure determination, pharmaceutical formulation, active pharmaceutical ingredients

## Abstract

3D electron diffraction (3DED) is increasingly employed to determine molec­ular and crystal structures from micro-crystals. Indomethacin is a well known, marketed, small-molecule non-steroidal anti-inflammatory drug with eight known polymorphic forms, of which four structures have been elucidated to date. Using 3DED, we determined the structure of a new ninth polymorph, σ, found within an amorphous solid dispersion, a product formulation sometimes used for active pharmaceutical ingredients with poor aqueous solubility. Subsequently, we found that σ indomethacin can be produced from direct solvent evaporation using di­chloro­methane. These results demonstrate the relevance of 3DED within drug development to directly probe product formulations.

## Abstract 2

3D electron diffraction (3DED) was used to elucidate the structure of a new ninth polymorph of indomethacin from an amorphous solid dispersion, which are product formulations used to improve the dissolution performance of active pharmaceutical ingredients with poor aqueous solubility. Insights from the structure solution allowed for a simpler crystallization route for this polymorph to be deduced, demonstrating the relevance of 3DED within drug development.

## 1. Introduction

Interest in 3D electron diffraction (3DED) techniques for structure solution from micro-crystals has increased rapidly within drug discovery over the past 5 years (Gemmi *et al.*, 2019 ▸; Jones *et al.*, 2018 ▸). 3DED (also known as MicroED) enables the determination of molecular structures from micro-crystals, eliminating the need for the larger crystals required for single-crystal X-ray diffraction (SCXRD) – the current gold standard for structure solution in the pharmaceutical industry – and unlocks possibilities for high-throughput structure determination. The potential impact of 3DED within drug development has been less prominent (Lightowler *et al.*, 2024 ▸): the majority of compounds with previously unknown structures that have been solved were of a single phase, studied in isolation; although there has been success with determining structures from off-the-shelf products (Jones *et al.*, 2018 ▸; Karothu *et al.*, 2023 ▸; Gruene *et al.*, 2018 ▸). We present here a 3DED study on a type of sample that may be investigated at the product formulation stage of development, with the added complications of an unexpected form of the active pharmaceutical ingredient (API) and the presence of an excipient.

**Figure 1:**
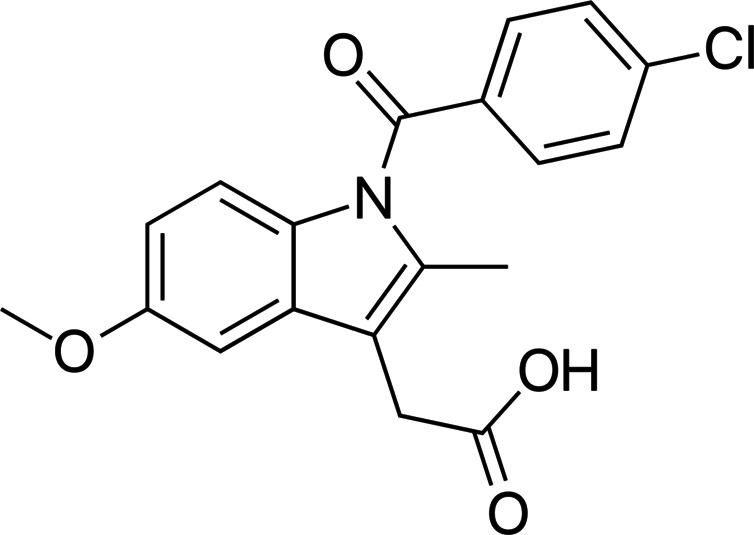
Indomethacin molecule C_19_H_16_ClNO_4_.

Indomethacin (Fig. 1 ▸) is a non-steroidal anti-inflammatory drug known to have eight polymorphic forms (Table 1 ▸). Despite having been widely used for over 40 years, the structures of several polymorphs (δ and θ) have only recently been revealed using 3DED (Lightowler *et al.*, 2022 ▸; Andrusenko *et al.*, 2021 ▸). Indomethacin is classified using the Biopharmaceutics Classification System (BCS) as class II (Butler & Dressman, 2010 ▸), indicating its poor aqueous solubility resulting in low oral bioavailability. Amorphous solid dispersions (ASDs) are a popular formulation strategy used to create superior dissolution performance – often referred to as the ‘spring effect’ – of poorly soluble pharmaceutical molecules by creating a stable solid dispersion of the API within an amorphous water-soluble polymer, which acts as an excipient (Vasconcelos *et al.*, 2007 ▸). Furthermore, ASDs aim to provide kinetic stability to prevent subsequent crystallization of the supersaturated drug within the gastrointestinal tract, often referred to as the ‘parachute effect’ (Hu *et al.*, 2019 ▸; Guzmán *et al.*, 2007 ▸). However, ASDs may experience storage life complications caused by unwanted phase separation and crystallization of the API which may affect the performance of the drug product, subject to different storage conditions such as humidity and temperature (Ricarte *et al.*, 2019 ▸; Xie & Taylor, 2017 ▸). Undesirable crystallinity found in ASDs may take the form of different polymorphs (S’ari *et al.*, 2021 ▸). These can be difficult to isolate from the formulation in the form of a single crystal of sufficient quality for SCXRD. This may be further exacerbated by the presence of multiple polymorphs, resulting in potential concern later on in the drug development process where the appearance of new forms can have serious consequences (Newman & Wenslow, 2016 ▸).

**Figure 2:**
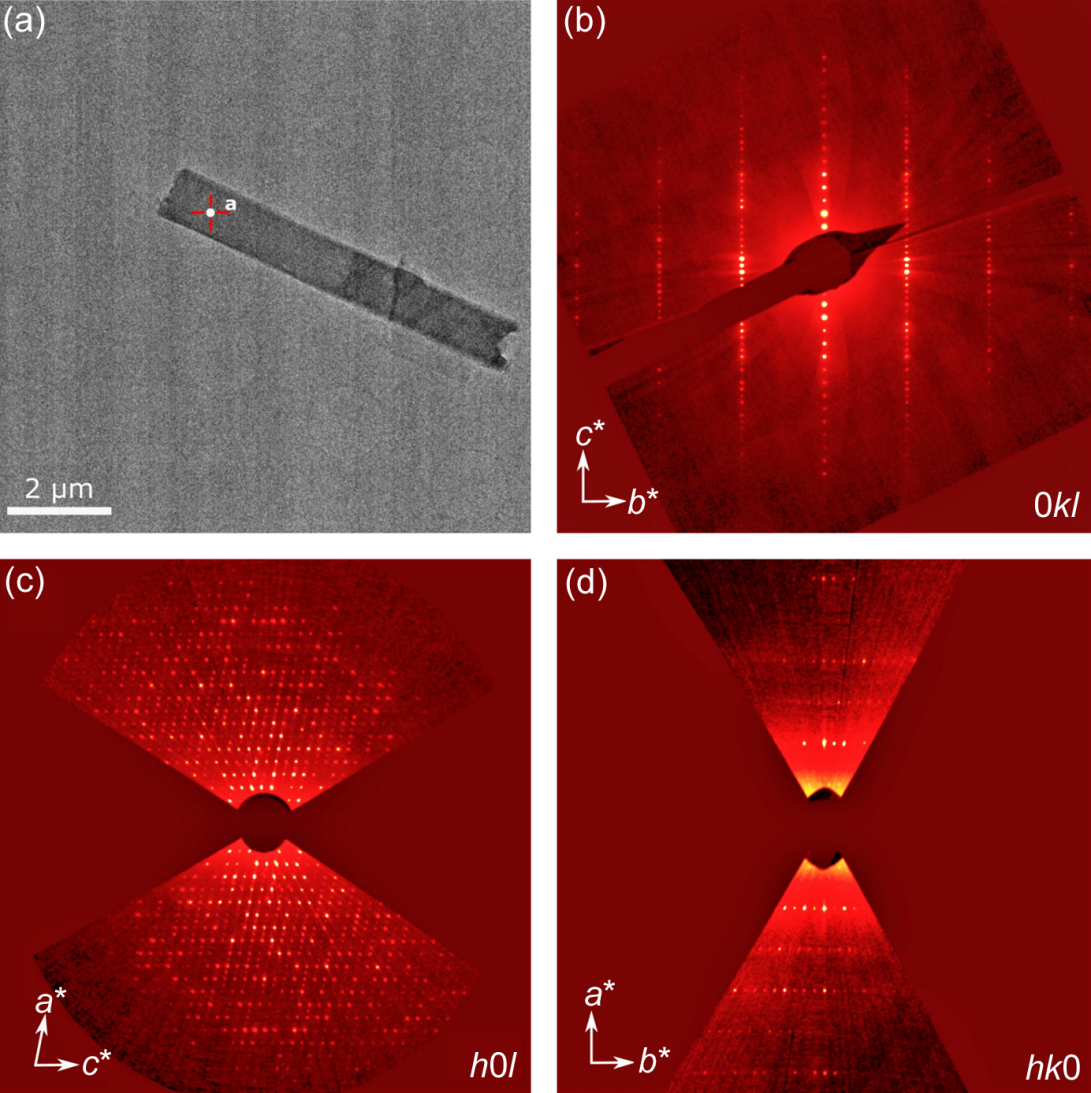
(*a*) Crystal from which structure solution was successful. It is positioned such that the [100]* direction is parallel to the direction of the electron beam at 0 ° tilt, based on the calculated orientation matrix. (*b*)–(*d*) Slices of reciprocal lattice planes from the reconstructed reciprocal space for indomethacin. Systematic absences suggest a monoclinic space group of *C*2/*c* or *Cc*.

In this work, we formulate an ASD of indomethacin and polyvinyl­pyrrolidone (PVP), prepared via the solvent-evaporation method using di­chloro­methane (DCM) (Ricarte *et al.*, 2019 ▸). We find crystallinity in the form of a new polymorph of indomethacin which grows in a whisker-like (∼30 nm wide) or lath-like (approximately several hundred nanometres wide) morphology [see Fig. 2 ▸(*a*)]. We solved the structure of this new polymorph using 3DED.

## 2. Methods

The presence of a new crystal form in the ASD was first identified using powder X-ray diffraction (PXRD). As expected, indomethacin (purchased from Sigma–Aldrich) was as polymorph γ, the most stable form. Given that a range of drug loadings are commonly found in commercial ASDs (He & Ho, 2015 ▸), ASDs across a range of drug loadings were produced using the solvent-evaporation method (see Section S1.1 of the supporting information). ASDs with loadings of 20:80 to 80:20 indomethacin:PVP appeared amorphous using PXRD. However, PXRD from 95:5 indomethacin:PVP ASDs feature a set of low-angle characteristic peaks which could not be matched to any of the polymorphs of indomethacin in the Cambridge Structural Database (CSD), but instead showed very strong similarities to the τ structure previously reported by Van Duong *et al.* (2018 ▸) in an indomethacin:polyethyl­ene glycol solid dispersion. Owing to the difficulty isolating single crystals of τ for SCXRD, the structure of this polymorph remains unsolved. For our structure, indexing the PXRD data was challenging, providing many possible monoclinic unit cells and space group options. Before turning to 3DED, several attempts to progress with structure determination from PXRD data via global optimization methods using some of the many candidate unit cells of our structure proved unsuccessful.

The ASD was deposited as a crushed powder onto Quantifoil R1.2/1.3 grids. 3DED data were collected from crystals several micrometres in size under cryogenic conditions using a Thermo Fisher Titan Krios G3i operated at 300 kV and a CETA-16M camera, revealing a monoclinic unit cell. Based on observed systematic absences, the search was narrowed to two possible space groups: *C*2/*c* or *Cc*. Though the occurrence of *Cc* structures in the CSD is rarer than *C*2*/c*, structure solution using both space groups was attempted before further conclusions were drawn.

A dataset obtained from one crystal [shown in Fig. 2 ▸(*a*)] was of sufficient quality to proceed with the structure solution, giving a completeness of 77% up to 0.8 Å resolution. The monoclinic cell parameters were found to be *a* = 43.70 (12) Å, *b* = 5.19 (7) Å, *c* = 33.43 (7) Å, *β* = 100.73 (9)°, *V* = 7448 (104) Å^3^. From the orientation matrix, the short crystallographic *b* axis is parallel to the long axis of the crystals, as might be expected from Bravais–Friedel–Donnay–Harker (Donnay & Harker, 1937 ▸) considerations. Crystals were found to lie with the (100) face flat on the grids [Fig. 2 ▸(*a*)]. Due to this preferred orientation of the crystals, a problem documented in 3DED (Lightowler *et al.*, 2022 ▸; Woollam *et al.*, 2020 ▸), it was not possible to collect to a completeness of greater than 80% even with the merging of multiple crystals. Given the sufficient quality of an individual dataset, we decided not to merge data from multiple crystals.

## 3. Results and discussion

The structure was successfully solved via the *ab initio* dual space method implemented in *SHELXD* (Schneider & Sheldrick, 2002 ▸) using the *C*2/*c* space group with two indomethacin molecules per asymmetric unit (*Z*′ = 2). All non-hydrogen atoms were found in the initial *C*2/*c* structure solution. Hydrogen atoms were generated in geometrically idealized positions. By comparison, structure solution using the *Cc* space group only yielded a partial solution. The positions of atoms found in the incomplete *Cc* solution matched the molecular conformation of those in the *C*2/*c* solution, but with an origin shift. However, refinement of the partial solution was highly unstable even with restraints to control the pseudosymmetry. Therefore, a final refinement was carried out using the least-squares methods in *SHELXL* for the *C*2/*c* space group with electron scattering factors [International Tables Vol. C: Tables 4.2.6.8 and 6.1.1.4]. Restraints were limited to maintaining the same geometry in the two independent molecules: there was no need for any further geometric or atomic displacement restraints. Isotropic atomic displacement factors were preferred because anisotropic refinement was not found to significantly improve the model. The final model gave an *R* factor of 28.22% refined using data to 0.8 Å resolution. Table S1 of the supporting information summarizes all relevant crystallographic information.

ED structures typically have higher *R* factors than those solved using SCXRD (Klar *et al.*, 2023 ▸). In this case, this is likely due to several factors. Both the structure solution and the refinement have been carried out using kinematical approximations assuming intensities are proportional to the square of the structure factor (*I*_*hkl*_ ∝ |*F*_*hkl*_|^2^); dynamical effects (*i.e.* multiple scattering) which will affect the observed intensities are not considered here (Palatinus *et al.*, 2015 ▸). Another factor is the induced radiation damage to the sample. This is qualitatively described by the observation of higher-order spots reducing in intensity across the tilt series. Quantitative modelling of the effects of electron beam irradiation damage are not yet well established (Peet *et al.*, 2019 ▸). The CETA camera used is also less sensitive compared with a direct electron detector and required a higher dose (a cumulative dose of 20 e Å^−2^ per tilt series), thus exacerbating sample damage and accurate detection of intensities. Finally, no correction was made to account for the effects of inelastic scattering.

**Figure 3:**
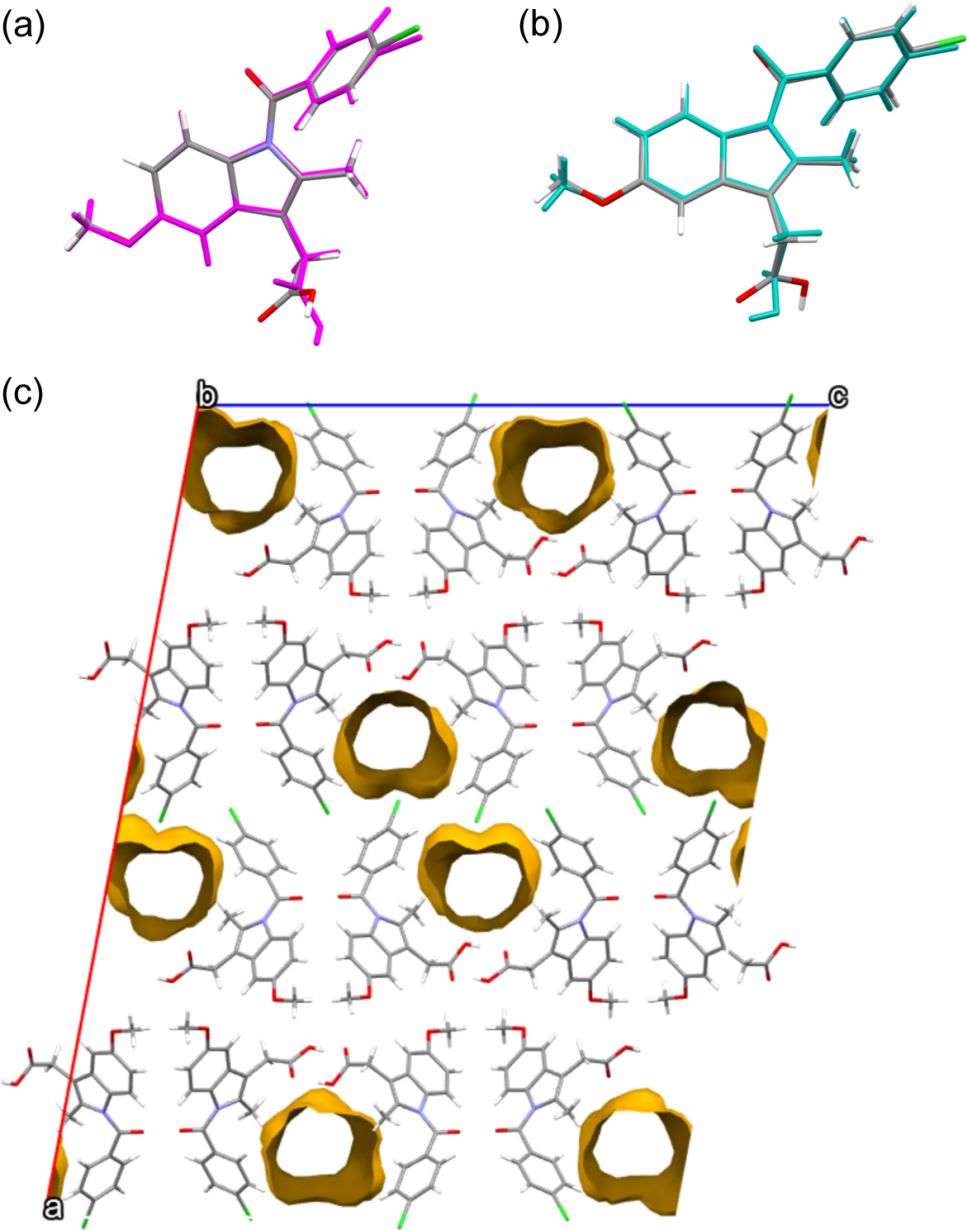
(*a*) Comparison between the two independent molecules of σ showing their conformational similarity (RMSD = 0.5003 Å). The biggest difference comes from the torsion angle of the carboxyl groups. Overlaying all atoms except the carb­oxy­lic acid oxygens leads to RMSD = 0.125 Å. (*b*) Conformational comparison between one of the indomethacin molecules in σ with one of the independent molecules in the δ structure (RMSD = 0.392 Å). Similarly to (*a*), the difference comes from the torsion angle of the carboxyl groups. Overlaying all atoms except the carb­oxy­lic acid oxygens leads to RMSD = 0.153 Å. (*c*) Yellow graphics highlight the channels in the σ structure which suggest that solvent templating was the mechanism for formation.

The asymmetric unit of the new polymorph contains two indomethacin molecules. The root mean square deviation (RMSD) – a measure of the average distance between the atoms of two superimposed molecules – between the indomethacin molecules is 0.5003 Å. As can be seen in Fig. 3 ▸(*a*), the torsion angles in the carboxyl groups of the different indomethacin molecules here are orthogonal but otherwise the molecules are highly similar. By considering the overlay for all non-hydrogen atoms except the carb­oxy­lic acid oxygens, the RMSD is reduced to 0.125 Å. The independent indomethacin molecules in this polymorph form a carb­oxy­lic dimer which is consistent with interactions found in the α, γ, θ and δ forms.

To confirm our 3DED model and show consistency with the bulk sample, a Rietveld rigid-body refinement, starting from an idealized model of indomethacin molecular geometry with five rotatable bonds and positions obtained from 3DED data, was performed on synchrotron PXRD data (Fig. S3 of the supporting information). This converged with a satisfactory fit (*R*_wp_ = 1.40%, χ^2^ = 2.09) without significant modification of the structure. Although characteristic peak positions from the new structure are very similar to that of the τ polymorph identified by Van Duong *et al.* (2018 ▸), the relative intensities of the peaks do not match, and this cannot be accounted for by the effects of texture. Most notably, the very weak intensity of the (400) peak in the new PXRD data contrasts with the strong peak at the corresponding location in τ. This cannot be explained by preferred orientation because the lower-order peak (200) is the strongest peak detected in the new structure: this is more than 8000 times stronger than (400), as shown in Fig. S4. The difference in observed intensities in PXRD data must instead come from differences within the unit cells. It is not possible to see these peaks in the ED data because these low-order reflections are covered by the shadow of the beam stop, which acts to protect the electron detector from the direct beam. However, given the similar characteristic peak positions, the τ polymorph and our structure are likely to be closely related and share strong structural similarities. We propose to name the new structure σ, the ninth polymorph of indomethacin.

On further inspection of σ, we note that 12% of the unit-cell volume is composed of predominantly hydro­phobic open channels parallel to the *b* axis [Fig. 3 ▸(*c*)]. Large void spaces in crystal structures can be associated with metastable phases (Kitaigorodskii, 1965 ▸; Barbas *et al.*, 2018 ▸; Sundareswaran & Karuppannan, 2020 ▸). The metastable nature of polymorph σ relative to the most thermodynamically stable γ phase is supported by the emergence of new peaks corresponding to the latter phase in the PXRD data collected around 10 months after the production of σ. Based on the spatial distribution of the channels, we considered the possibility that a smaller molecule (such as solvent) is responsible for the empty space in the structure and is relevant to the crystallization mechanism of σ. We propose that DCM molecules (the solvent used) may have acted as a backbone solvent template, running in channels through the structure during solvent evaporation as crystals formed. The total channel volume in the unit cell is 903 Å^3^, with 8 channels in each unit cell. The molar volume of DCM is ∼68 Å^3^, suggesting that each channel could fit two solvent molecules given that we would expect channels in the structure to decrease in volume upon desolvation. The volatility of DCM makes it highly unlikely to remain within the indomethacin structure on drying. This is further reinforced by the lack of residual electron density observed in the channels. To confirm this hypothesis, evaporation of a DCM solution of pure indomethacin was carried out (on a crystallization dish left at room temperature, with no additional heating or pressure necessary), also yielding the σ structure observed using PXRD. This is consistent with the theory of crystallization via solvent templating (Gnutzmann *et al.*, 2014 ▸; Klimakow *et al.*, 2010 ▸).

Consequently, based on our observations from the solved structure, we have developed a simpler route to forming this polymorph. Although DCM is not a solvent generally used in manufacturing processes within the pharmaceutical industry owing to its environmental impact, the more open structure of this metastable form of indomethacin may be of potential interest as it will likely have different dissolution properties compared with the most thermodynamically stable γ phase. A similar theory was proposed for the crystallization of the δ polymorph, whereby the indomethacin methanol solvate first crystallizes before desolvation (Andrusenko *et al.*, 2021 ▸). However, the methanol directly disrupts the hydrogen bonding between indomethacin molecules, whereas in σ the positions of the channels suggest that the DCM molecules do not interact strongly with indomethacin, as would be expected based on its lack of hydrogen bond donors and acceptors. Interestingly, although σ and δ have different packing arrangements, the conformations of the individual indomethacin molecules are broadly comparable [an example of this conformational fit is shown in Fig. 3 ▸(*b*)].

To conclude, we have isolated a metastable phase of indomethacin from an amorphous solid dispersion and reported the structure of a new polymorph (which we call σ) using 3DED. The solution obtained suggested a crystallization mechanism of solvent templating and provided an easier experimental route to the new form via evaporation of pure indomethacin from DCM. This demonstrates the applicability of 3DED to drug development in addition to drug discovery.

## 4. Related literature

The following references are cited in the supporting information: Coelho (2018 ▸); Macrae *et al.* (2020 ▸); Pham *et al.* (2010 ▸); Rigaku (2020 ▸).

## Supplementary Material

Crystal structure: contains datablock(s) I. DOI: 10.1107/S2052252524008121/of5005sup1.cif

Structure factors: contains datablock(s) indomethacin_sigma. DOI: 10.1107/S2052252524008121/of5005sup2.hkl

Input file from the Rietveld Refinement carried out on the PXRD data. DOI: 10.1107/S2052252524008121/of5005sup3.txt

Supporting figures and table. DOI: 10.1107/S2052252524008121/of5005sup4.pdf

CCDC reference: 2355453

## Figures and Tables

**Table 1:** Summary of the known polymorphic forms of indomethacin to date

Polymorph	Year discovered	Year solved	CSD identifier	Method
γ	1968	1972†	INDMET (01, 03, 05, 06)	SCXRD
α	1968	2002‡	INDMET (02, 04)	SCXRD
δ	1998	2021§¶	INDMET (07, 08)	3DED
ɛ	2013§	–	–	–
η	2013§	–	–	–
ζ	2013§	–	–	–
τ	2018[Table-fn tfn2]	–	–	–
θ	2022	2022¶	INDMET (09)	3DED

† Surwase *et al.* (2013 ▸).

‡ Van Duong *et al.* (2018 ▸).

§ Kistenmacher & Marsh (1972 ▸).

¶ Chen *et al.* (2002 ▸).

†† Andrusenko *et al.* (2021 ▸).

‡‡ Lightowler *et al.* (2022 ▸).

## Data Availability

The authors confirm that the data supporting the findings of this study are available within the article and its supplementary materials.
