# Supplementary material for: From formulation to structure: 3D electron diffraction for the structure solution of a new indomethacin polymorph from an amorphous solid dispersion
Source: IUCrJ. 2024 Aug 28;11(Pt 5):744–8. doi: 10.1107/S2052252524008121 (PMC11364028; doi:10.1107/S2052252524008121)
Supplement: Supplementary file 4 [file m-11-00744-sup4.pdf]

IUCrJ

Volume 11 (2024)

Supporting information for article:

From Formulation to Structure: 3D Electron Diffraction for Structure Solution of a New Indomethacin Polymorph from an Amorphous Solid Dispersion

Helen W. Leung, Royston C. B. Copley, Giulio I. Lampronti, Sarah J. Day, Lucy K. Saunders, Duncan N. Johnstone and Paul A. Midgley

S1. Experimental Procedures

S1.1. Solvent Evaporation

Amorphous solid dispersions (ASDs) of indomethacin and polyvinylpyrrolidone (PVP) were prepared using the solvent evaporation method as described by Pham et al. (Pham *et al.*, 2010). Indomethacin and PVP K25, purchased from Sigma Aldrich, were weighed to make up a combined mass of 0.5 g. ASDs with a range of drug loadings from 20:80 wt% to 95:5 wt% indomethacin/PVP were made. The powder was dissolved in approximately 10 cm^3 of dicholoromethane to form a yellow solution. A rotary evaporator was used to boil off the dichloromethane solvent which was placed in a water bath at 40 °C under a pressure of 40 mBar. The resulting powder coated the glass vial which was then placed in a vacuum overnight to ensure any remaining solvent was evaporated. Prior to use, each sample was stored at room temperature in a dessicator.

S1.2. X-Ray Powder Diffraction

In-situ XRPD characterisation was performed at Diamond beamline I11. ASD powder was loaded into a 0.5 mm borosilicate glass capillary and analysed using an X-ray beam of 0.82408 Å wavelength (15 keV energy), refined using a NIST SRM640c Si standard. The sample was cooled to 80 K, consistent with 3D-ED measurement conditions, using a Cryostream Plus. Measurements were made using the Mythen wide-angle position sensitive detector (PSD).

S1.3. 3D-ED sample preparation and data collection

3D-ED was performed under cryogenic conditions using a Thermo Fisher Titan Krios G3i operated at 300 kV and a CETA-16M camera. A diffraction pattern was recorded for each tilt increment over a range of ± 60 ° where the relative position of the crystal of interest with respect to the grid bars or other crystals would allow at a continuous tilt rate of 1 °/s, with an exposure time of 0.5 s per frame, leading to 240 frames. EPU-D software was used for this acquisition, and the auto-eucentric height function was used to minimise sample movement when tilting to high angles. Despite this correction, 3D-ED could only be performed on the larger lath-like crystals due to the significant relative image movement of the whisker-like crystals.

3D-ED data were recorded from 10 different crystals with sizes ranging between 100 nm to several microns. EPU-D software was used to capture information relating to crystal morphologies in low magnification mode on the TEM to easily direct us to crystals that were ideally positioned and isolated. Camera lengths were set such that the edge of the detector allowed for a resolution of 0.75 Å. Well-isolated crystals which displayed little evidence of significant mosaicity (as observed in diffraction mode) gave clean diffraction patterns and were selected for further data analysis.

S2. Results and Discussion

S2.1. 3D-ED Data Processing

3D-ED data were analysed using Rigaku CrysAlisPro 1.171.41.93a) software (Rigaku Oxford Diffraction, 2020), which allowed for reciprocal space reconstruction and visualisation, cell parameter determination, and reflection intensity integration. This analysis revealed consistent symmetry and lattice constants between different crystals. Out of 10 datasets deemed high enough quality for further analysis, reciprocal reconstruction revealed one of these crystals was the (already solved) α phase. The rest were consistent with the monoclinic unit cell of the σ polymorph and were used to confirm unit cell parameters.

The best dataset was used for structure determination and refinement with the *C*2/*c* space group. The Olex2 interface was used to access SHELXD and SHELXL for structure solution and refinement respectively. Refined structures were subsequently analysed using Mercury software (MacRae *et al.*, 2020), taking advantage of its tools for conformational comparison between molecules and void calculation.

S2.2. Rietveld Refinement from X-Ray Powder Diffraction

With a crystal structure of σ indomethacin having been obtained from 3D-ED methods, we returned to XRPD to ensure the validity of the structure by carrying out refinement using the 3D-ED model. Rietveld refinement was carried out using TOPAS academic software (Coelho, 2018). The measurement of an empty capillary was used as an empirical background with a scale factor which was set as a refinable parameter. On top of this, the background was further described with a 6-parameter Chebyshev function. Pawley fitting showed significant anisotropy which was modelled with spherical harmonics and strain. The indomethacin molecule was modelled as a rigid body with the same positions and orientations as in the σ molecules where the torsion angles associated with the five rotatable bonds were freely refined based on its idealised initial geometry from the γ structure solved by SCXRD (CCDC ref 1180373). The refinement converged to R_{wp} = 1.40% and χ^2 = 2.09.

S3. Supporting Tables

Table S1 Selected parameters from the structure refinement using SHELXL, generated by XCIF. The structure solution and refinement executed here makes use of workflows which come from X-ray crystallography protocols. As such, we recognise that some parameters are not ideal for use with electron diffraction. For example, the semi-empirical absorption corrections here likely account for effects of beam damage whilst small Bragg angles associated with electron diffraction data likely contribute to larger EXTI parameters (and larger errors in this value). However, refinement without these semi-empirical corrections led to a worse model and higher R-factor. *Number of electrons in the unit cell.

Empirical formula	C_{19} H_{16} Cl N O_4	
Formula weight	357.79	
Temperature	87(2) K	
Wavelength	0.0197 Å	
Crystal system	Monoclinic	
Space group	C 2/c	
Unit cell dimensions	a = 43.70(12) Å	α= 90°.
	b = 5.19(7) Å	β= 100.73(9)°.
	c = 33.43(7) Å	γ = 90°.
Volume	7448(104) Å^3	
Z	16	
Density (calculated)	1.276 Mg/m^3	
Absorption coefficient	0.000 mm^{-1}	
F(000)	1114*	
Crystal size	0.0070 x 0.0010 x 0.0001 mm^3	
Theta range for data collection	0.057 to 0.705°.	
Index ranges	-48<=h<=47, -6<=k<=6, -41<=l<=41	
Reflections collected	15410	
Independent reflections	5885 [R(int) = 0.1371]	
Absorption correction	Semi-empirical from equivalents	
Max. and min. transmission	1.00000 and 0.01541	
Refinement method	Full-matrix least-squares on F^2	
Data / restraints / parameters	5885 / 66 / 208	
Goodness-of-fit on F^2	1.165	

Final R indices [I>2sigma(I)]	R1 = 0.2822, wR2 = 0.6107
R indices (all data)	R1 = 0.3254, wR2 = 0.6347
Extinction coefficient	534(71)
Largest diff. peak and hole	0.452 and -0.423 e.Å^{-3}

S4. Supporting Figures

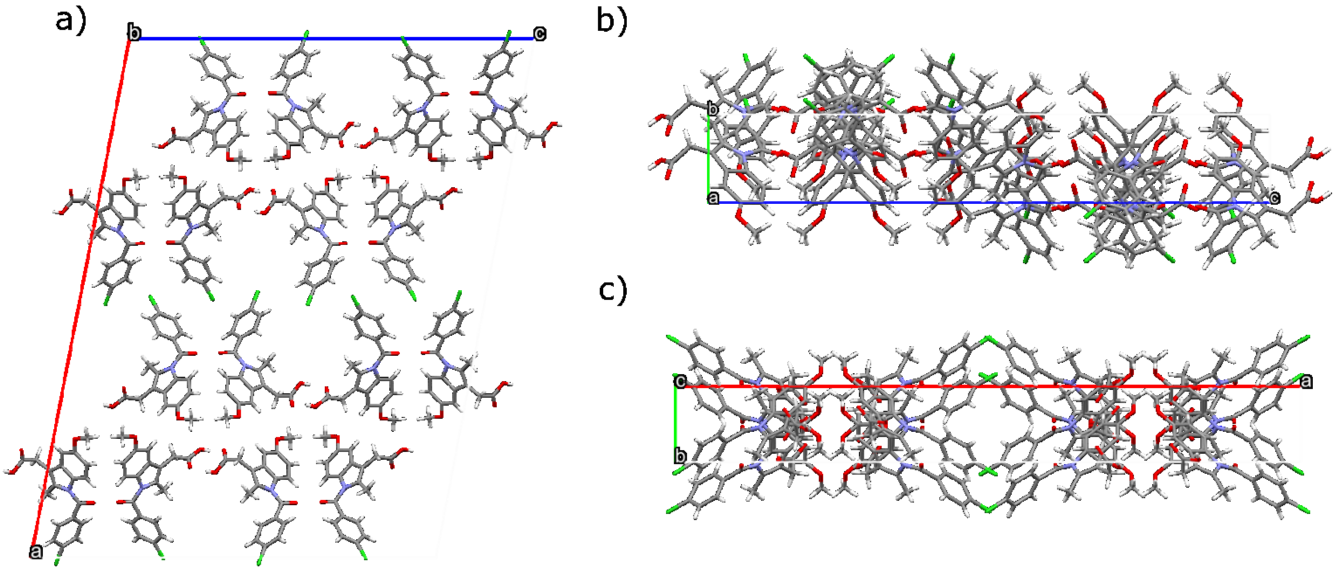

Figure S1 Molecular packing of the σ indomethacin structure viewed along a) *b*-axis, b) *a*-axis, c) *c*-axis.

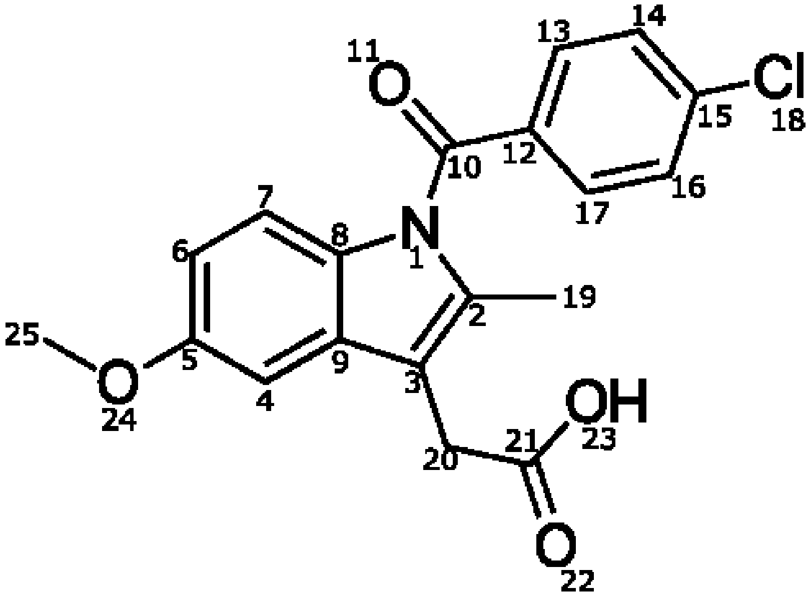

Figure S2 Labels used for structure refinement of indomethacin. The 2nd indomethacin molecule in the asymmetric unit has identical labels, except starting from N50 (50 is added to all numbers).

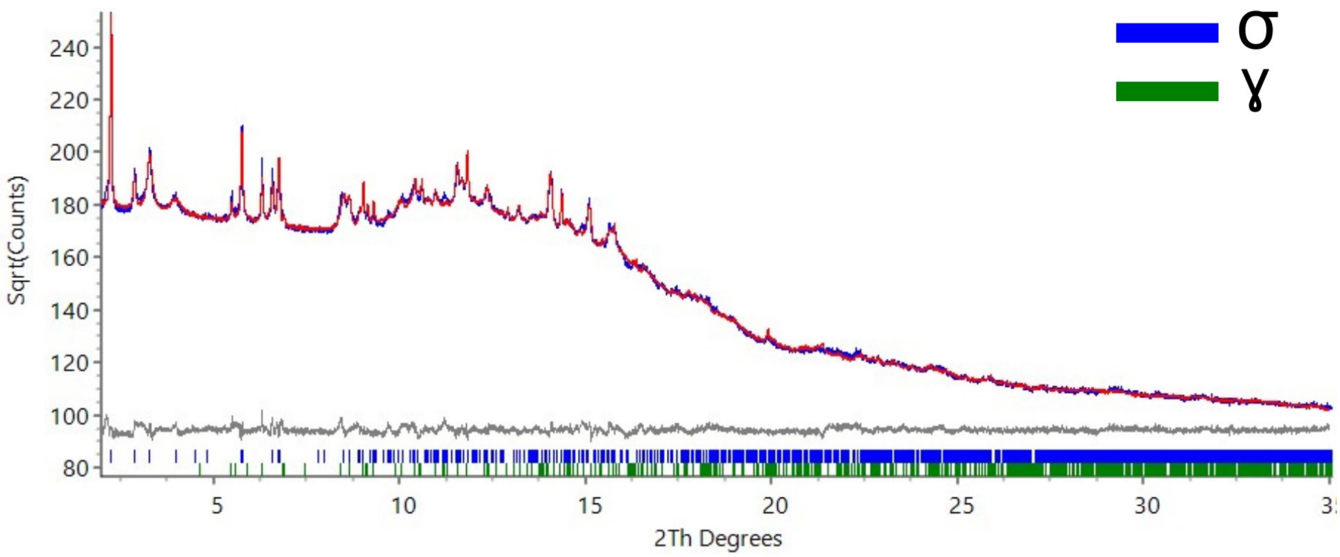

Figure S3 Rietveld Refinement of high resolution XRPD data using the structural model obtained from 3D-ED. This shows consistency between the bulk powder and single crystal data. R_{wp} = 1.40% and χ^2 = 2.09. The presence of the γ phase is also modelled in this refinement.

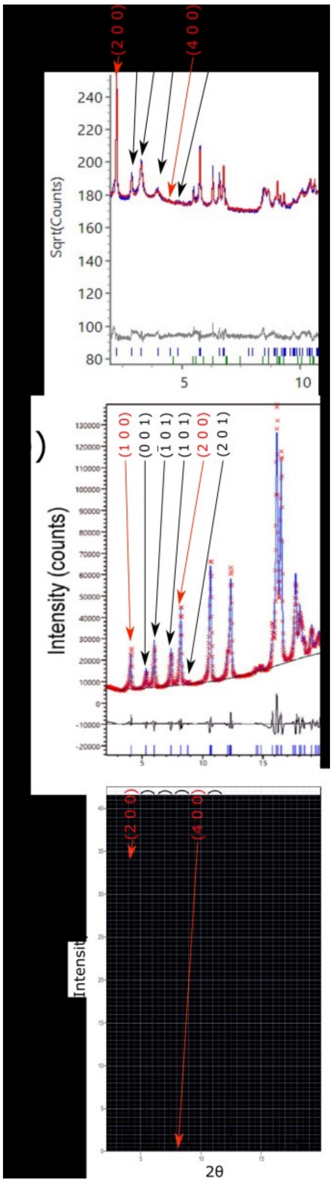

Figure S4 A comparison of the XRPD trace from (a) our σ structure (λ = 0.82408 Å), (b) the unsolved τ structure (λ = 1.54060 Å) (Van Duong *et al.*, 2018), and (c) our σ structure (simulated at λ = 1.54060 Å for easier comparison as the differences in wavelength used for experiments means the experimental peak positions cannot be directly overlaid). Furthermore, a different proposed unit cell leads to different indexing of the resulting peaks. However, we point to the differences in relative intensities in (a) and (c) between the $(2\ 0\ 0)_{\sigma}$, the strongest peak, and $(4\ 0\ 0)_{\sigma}$, a peak almost absent in our structure compared to in (b) the corresponding $(1\ 0\ 0)_{\tau}$ and $(2\ 0\ 0)_{\tau}$ peaks, where the $(2\ 0\ 0)_{\tau}$ peak in τ is even stronger than $(1\ 0\ 0)_{\tau}$. This cannot be explained by effects of texture because related parallel crystal planes (e.g. $(2\ 0\ 0)_{\sigma}$, $(4\ 0\ 0)_{\sigma}$ and $(1\ 0\ 0)_{\tau}$, $(2\ 0\ 0)_{\tau}$ should be affected in the same way by preferred orientation. *(b) Reprinted (adapted) with permission from {Tu Van Duong et al., Molecular Pharmaceutics 2018 15 (3), 1037-105, 10.1021/acs.molpharmaceut.7b00930}. Copyright {2018} American Chemical Society.*

References

Andrusenko, I., Hamilton, V., Lanza, A. E., Hall, C. L., Mugnaioli, E., Potticary, J., Buanz, A., Gaisford, S., Piras, A. M., Zambito, Y., Hall, S. R. & Gemmi, M. (2021). *Int. J. Pharm.* **608**, 121067.

Barbas, R., Font-Bardia, M. & Prohens, R. (2018). *Cryst. Growth Des.* **18**, 3740–3746.

Butler, J. M. & Dressman, J. B. (2010). *J. Pharm. Sci.* **99**, 4940–4954.

Chen, X., Morris, K. R., Griesser, U. J., Byrn, S. R. & Stowell, J. G. (2002). *J. Am. Chem. Soc.* **124**, 15012–15019.

Coelho, A. A. (2018). *J. Appl. Crystallogr.* **51**, 210–218.

Donnay, J. D. H. & Harker, D. (1937). *Am. Mineral.* **22**, 446–467.

Van Duong, T., Lüdeker, D., Van Bockstal, P. J., De Beer, T., Van Humbeeck, J. & Van Den Mooter, G. (2018). *Mol. Pharm.* **15**, 1037–1051.

Gemmi, M., Mugnaioli, E., Gorelik, T. E., Kolb, U., Palatinus, L., Boullay, P., Hovmö, S. & Abrahams, J. P. (2019). *ACS Cent. Sci.* **5**, 1315–1329.

Gnutzmann, T., Nguyen Thi, Y., Rademann, K. & Emmerling, F. (2014). *Cryst. Growth Des.* **14**, 6445–6450.

Gruene, T., Wennmacher, J. T. C., Zaubitzer, C., Holstein, J. J., Heidler, J., Fecteau-Lefebvre, A., De Carlo, S., Müller, E., Goldie, K. N., Regeni, I., Li, T., Santiso-Quinones, G., Steinfeld, G., Handschin, S., van Genderen, E., van Bokhoven, J. A., Clever, G. H. & Pantelic, R. (2018). *Angew. Chemie - Int. Ed.* **57**, 16313–16317.

Guzmán, H. R., Tawa, M., Zhang, Z., Ratanabanangkoon, P., Shaw, P., Gardner, C. R., Chen, H., Moreau, J. P., Almarsson, Ö. & Remenar, J. F. (2007). *J. Pharm. Sci.* **96**, 2686–2702.

He, Y. & Ho, C. (2015). *J. Pharm. Sci.* **104**, 3237–3258.

Hu, C., Hu, C., Liu, Z., Liu, C., Li, J., Wang, Z., Xu, L., Chen, C., Fan, H. & Qian, F. (2019). *Mol. Pharm.* **16**, 4978–4986.

Jones, C. G., Martynowycz, M. W., Hattne, J., Fulton, T. J., Stoltz, B. M., Rodriguez, J. A., Nelson, H. M. & Gonen, T. (2018). *ACS Cent. Sci.* **4**, 1587–1592.

Karothu, D. P., Alhaddad, Z., Göb, C. R., Schürmann, C. J., Bücker, R. & Naumov, P. (2023). *Angew. Chemie Int. Ed.* **62**, e202303761.

Kistenmacher, T. J. & Marsh, R. E. (1972). *J. Am. Chem. Soc.* **94**, 1340–1345.

Kitaigorodskii, A. I. (1965). *Acta Crystallogr.* **18**, 585–590.

Klar, P. B., Krysiak, Y., Xu, H., Steciuk, G., Cho, J., Zou, X. & Palatinus, L. (2023). *Nat. Chem. 2023 156* **15**, 848–855.

Klimakow, M., Leiterer, J., Kneipp, J., Rössler, E., Panne, U., Rademann, K. & Emmerling, F. (2010). *Langmuir* **26**, 11233–11237.

Lightowler, M., Li, S., Ou, X., Cho, J., Liu, B., Li, A., Hofer, G., Xu, J., Yang, T., Zou, X., Lu, M. & Xu, H. (2024). *Angew. Chemie Int. Ed.* **63**, e202317695.

Lightowler, M., Li, S., Ou, X., Zou, X., Lu, M. & Xu, H. (2022). *Angew. Chemie Int. Ed.* **61**, e202114985.

MacRae, C. F., Sovago, I., Cottrell, S. J., Galek, P. T. A., McCabe, P., Pidcock, E., Platings, M., Shields, G. P., Stevens, J. S., Towler, M. & Wood, P. A. (2020). *J. Appl. Crystallogr.* **53**, 226–235.

Newman, A. & Wenslow, R. (2016). *AAPS Open 2016 21* **2**, 1–11.

Palatinus, L., Corrêa, C. A., Steciuk, G., Jacob, D., Roussel, P., Boullay, P., Klementová, M., Gemmi, M., Kopeček, J., Domeneghetti, M. C., Cámara, F. & Petříček, V. (2015). *Acta Crystallogr. Sect. B Struct. Sci. Cryst. Eng. Mater.* **71**, 740–751.

Peet, M. J., Henderson, R. & Russo, C. J. (2019). *Ultramicroscopy* **203**, 125–131.

Pham, T. N., Watson, S. A., Edwards, A. J., Chavda, M., Clawson, J. S., Strohmeier, M. & Vogt, F. G. (2010). *Mol. Pharm.* **7**, 1667–1691.

Ricarte, R. G., Van Zee, N. J., Li, Z., Johnson, L. M., Lodge, T. P. & Hillmyer, M. A. (2019). *Mol. Pharm.* **16**, 4089–4103.

Rigaku Oxford Diffraction (2020).

S'ari, M., Blade, H., Cosgrove, S., Drummond-Brydson, R., Hondow, N., Hughes, L. P. & Brown, A. (2021). *Mol. Pharm.* **18**, 1905–1919.

Schneider, T. R. & Sheldrick, G. M. (2002). *Acta Cryst D* **58**, 1772–1779.

Sundareswaran, S. & Karuppannan, S. (2020). *Cryst. Res. Technol.* **55**, 2000083.

Surwase, S. A., Boetker, J. P., Saville, D., Boyd, B. J., Gordon, K. C., Peltonen, L. & Strachan, C. J. (2013). *Mol. Pharm.* **10**, 4472–4480.

Vasconcelos, T., Sarmento, B. & Costa, P. (2007). *Drug Discov. Today* **12**, 1068–1075.

Woollam, G. R., Das, P. P., Mugnaioli, E., Andrusenko, I., Galanis, A. S., Van De Streek, J., Nicolopoulos, S., Gemmi, M. & Wagner, T. (2020). *CrystEngComm* **22**, 7490–7499.

Xie, T. & Taylor, L. S. (2017). *J. Pharm. Sci.* **106**, 100–110.
